# EBV-LMP1 promotes radioresistance by inducing protective autophagy through BNIP3 in nasopharyngeal carcinoma

**DOI:** 10.1038/s41419-021-03639-2

**Published:** 2021-04-01

**Authors:** San Xu, Zhuan Zhou, Xingzhi Peng, Xuxiu Tao, Peijun Zhou, Kun Zhang, Jinwu Peng, Dan Li, Liangfang Shen, Lifang Yang

**Affiliations:** 1 Department of Oncology, Key Laboratory of Carcinogenesis and Cancer Invasion of Ministry of Education, Xiangya Hospital, Central South University, Changsha, China; 2 Cancer Research Institute, School of Basic Medicine Science, Central South University, Changsha, China; 3 Department of Dermatology, Xiangya Hospital, Central South University, Changsha, China; 4 Department of Pathology, Xiangya Hospital, Central South University, Changsha, China; 5 Institute of Molecular Medicine and Oncology, College of Biology, Hunan University, Changsha, China; 6 Hunan Key Laboratory of Oncotarget Gene, Hunan Cancer Hospital and The Affiliated Cancer Hospital of Xiangya School of Medicine, Central South University, Changsha, China

**Keywords:** Oncogenes, Head and neck cancer

## Abstract

Studies have indicated that dysfunction of autophagy is involved in the initiation and progression of multiple tumors and their chemoradiotherapy. Epstein–Barr virus (EBV) is a lymphotropic human gamma herpes virus that has been implicated in the pathogenesis of nasopharyngeal carcinoma (NPC). EBV encoded latent membrane protein1 (LMP1) exhibits the properties of a classical oncoprotein. In previous studies, we experimentally demonstrated that LMP1 could increase the radioresistance of NPC. However, how LMP1 contributes to the radioresistance in NPC is still not clear. In the present study, we found that LMP1 could enhance autophagy by upregulating the expression of BCL2/adenovirus E1B 19 kDa protein-interacting protein 3 (BNIP3). Knockdown of BNIP3 could increase the apoptosis and decrease the radioresistance mediated by protective autophagy in LMP1-positive NPC cells. The data showed that increased BNIP3 expression is mediated by LMP1 through the ERK/HIF1α signaling axis, and LMP1 promotes the binding of BNIP3 to Beclin1 and competitively reduces the binding of Bcl-2 to Beclin1, thus upregulating autophagy. Furthermore, knockdown of BNIP3 can reduce the radioresistance promoted by protective autophagy in vivo. These data clearly indicated that, through BNIP3, LMP1 induced autophagy, which has a crucial role in the protection of LMP1-positive NPC cells against irradiation. It provides a new basis and potential target for elucidating LMP1-mediated radioresistance.

## Introduction

Nasopharyngeal carcinoma (NPC) is the most malignant tumor in the Southeast Asia region and south China. The primary clinical treatment of NPC is radiotherapy. However, radioresistance and metastasis affect the treatment outcomes and remain the key problems related to survival for NPC patients^1,2^. Epstein-Barr virus (EBV) is a lymphotropic human gamma herpesvirus which has been implicated in the pathogenesis of several human malignancies, including Burkitt’s and Hodgkin’s lymphomas, gastric carcinoma, and NPC^3^. EBV encoded latent membrane protein1 (LMP1) is thought to play a key role in the pathogenesis of NPC. It triggers several important signal transduction pathways, such as NF-κB, MAPK, JAK/STAT, PI3K/AKT, and others involved in the proliferation, apoptosis, and metastasis of tumor cells^4,5^. In previous studies, we experimentally demonstrated that LMP1 has an important role in the regulation of the radioresistance of NPC cells^6–8^. However, the mechanism of LMP1-mediated radioresistance is not entirely clear.

Autophagy is a conserved biological process for digestion and recycling of cytoplasmic constituents in eukaryotic cells, which is essential for maintaining genomic integrity and homeostasis^9^, and can be induced in response to various conditions including nutrient deprivation, metabolic stress, and hypoxia to adapt cellular conditions for survival^10^. Accumulating evidence has indicated that autophagy is important in the regulation of cancer development and progression, and in determining the response of tumor cells to anticancer therapies in a wide variety of tumors, including lung cancer, breast cancer, malignant glioma, and pancreatic cancer^11,12^. For example, in pancreatic cancer cells, defective SMAD4 is responsible for radioresistance through increased levels of radiation-induced autophagy^13^. The studies by Chen X et al. show that hypoxia enhanced cell radioresistance by increasing the induction of autophagy in non-small cell lung cancer^14^. These studies suggest that autophagy may play an important role in tumor radioresistance, although its mechanism remains unclear.

In the B lymphocytes, LMP1 induces autophagy in a dose-dependent manner and, thus, the inhibition of autophagy in EBV-positive cells leads to a decreased ability to form colonies^15^. Therefore, whether LMP1 is involved in the regulation of autophagy of NPC, and how LMP1-mediated autophagy plays a role in tumor radioresistance, needs further exploration. In this study, we found that LMP1 could enhance the autophagy of NPC cells, and the BCL2/adenovirus E1B 19 kDa protein-interacting protein 3 (BNIP3) was identified as a key modulator of LMP1-induced autophagy. Furthermore, the ERK/HIF1α signaling axis was confirmed to be crucial for LMP1-induced upregulation of BNIP3, and regulation of autophagy by LMP1-induced BNIP3 is in a Beclin1-dependent manner. These findings provide a potential strategy for the sensibilization therapy of LMP1-positive NPC, by using the inhibitor of autophagy or BNIP3.

## Results

### EBV-LMP1 induces autophagy in NPC cells

**Fig. 1:**
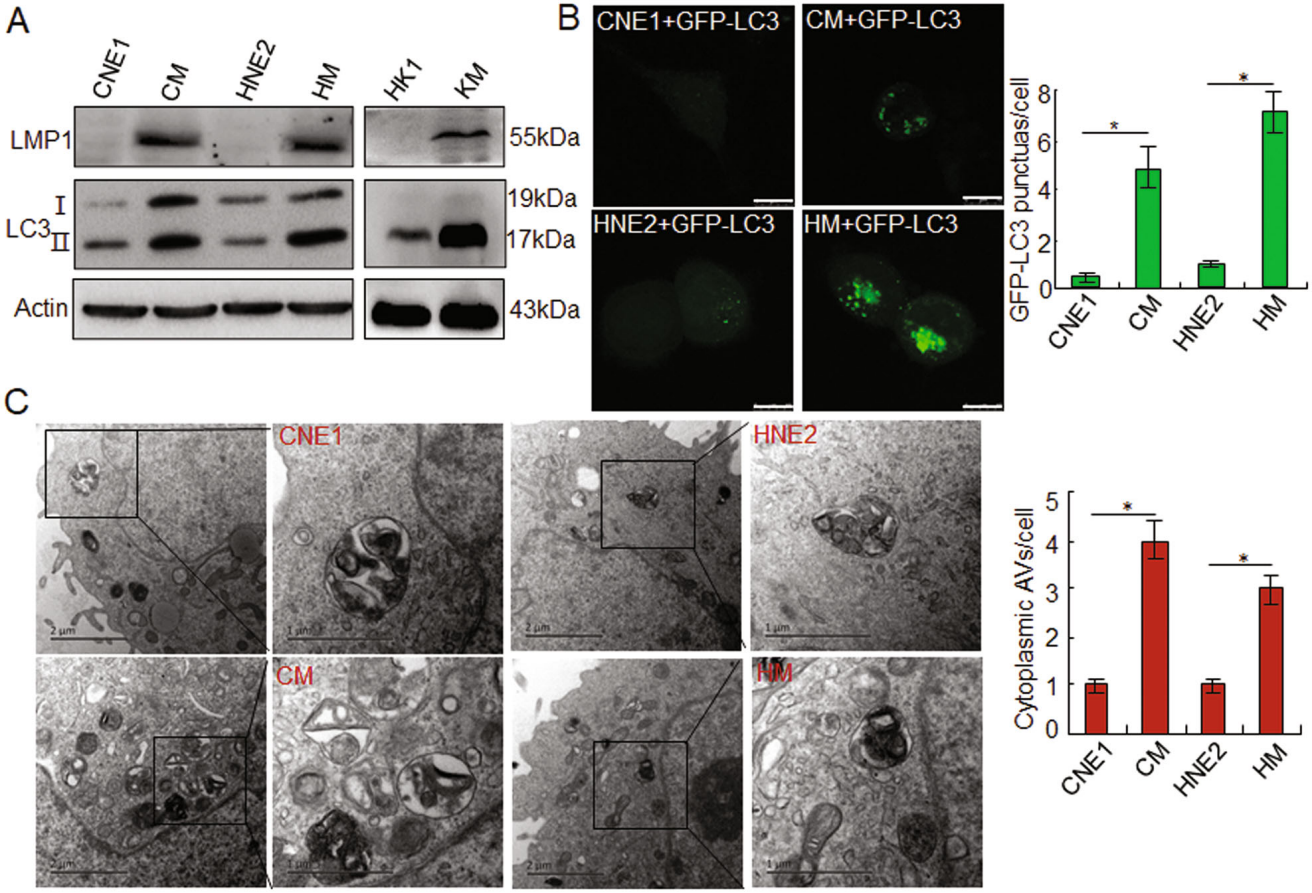
EBV-LMP1 induces autophagy in NPC cells. **A** The expression of LMP1 and LC3 I/II in CNE1, CM, HNE2, HM, HK1, and KM cells by Western blots. **B** After transfection with GFP-LC3 for 24 h, accumulation of GFP-LC3 puncta in NPC cells. Scale bar: 10 μm. **C** Transmission electron images of different NPC cells. Data are expressed as means ± S.D. of three experiments. *p < 0.05.

To investigate whether EBV-LMP1 induces autophagy in NPC cells, we used LMP1-positive cells, CM, HM and KM, and LMP1-negative cells, CNE1, HNE2, and HK1, for analysis. The data indicated that in the LMP1-positive cells, the LC3-II level was significantly increased compared to the LMP1-negative cells (Fig. 1A). Further, immunofluorescence experiments also found that transfection with GFP-LC3 plasmids resulted in a significant increase of LC3 puncta in CM and HM cells compared to CNE1 and HNE2 cells (Fig. 1B). Moreover, to further investigate the effects of LMP1 on autophagy, transmission electron microscopy was utilized. A greater number of double-membrane vesicles accumulated in the CM and HM cells compared to the control group (Fig. 1C). These results suggested that LMP1 could induce autophagy in NPC cells.

### LMP1 induces autophagy through BNIP3

**Fig. 2:**
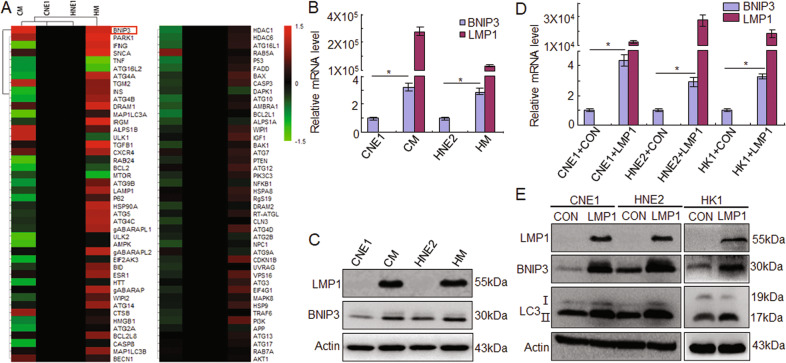
LMP1 increases BNIP3 expression in NPC cells. **A** A PCR array was used to detect changes in the expression of autophagy-related genes in LMP1-positive NPC cells compared with LMP1-negative NPC cells. **B** Quantitative RT-PCR and **C** Western blots were performed to analyze LMP1 and BNIP3 expression in NPC cells. **D** Quantitative RT-PCR and **E** Western blots were performed to analyze LMP1, BNIP3, and LC3 expression after transfection with LMP1 in NPC cells. CON: negative control, Data are expressed as means ± S.D. of three experiments. *p < 0.05.

To determine which molecules are involved in enhancing LMP-mediated autophagy in NPC cells, PCR-Array screening was applied to investigate the alteration of autophagy associated genes at the mRNA level in LMP1-positve CM cells in comparison with LMP1-negative CNE1 cells. The results showed that, among the 88 genes, the transcription level of BNIP3 was most obviously increased in the CM and HM cells compared to the CNE1 and HNE2 cells (Fig. 2A). To further confirm the upregulation of BNIP3 in LMP1-positive cells, the mRNA and protein level of LMP1 and BNIP3 were detected in the NPC cells. The data showed that BNIP3 expression in LMP1 positive NPC cells was significantly higher than in LMP1 negative cells at both the mRNA (*p* < 0.05) and protein level (Fig. 2B, C). After transient transfection with pGV141-LMP1-wt plasmids in CNE1,HNE2, and HK1 cells, the results indicated that LMP1 could induce BNIP3 and LC3II expression in NPC cells, suggesting LMP1 could induce autophagy related to BNIP3 (Fig. 2D, E).

**Fig. 3:**
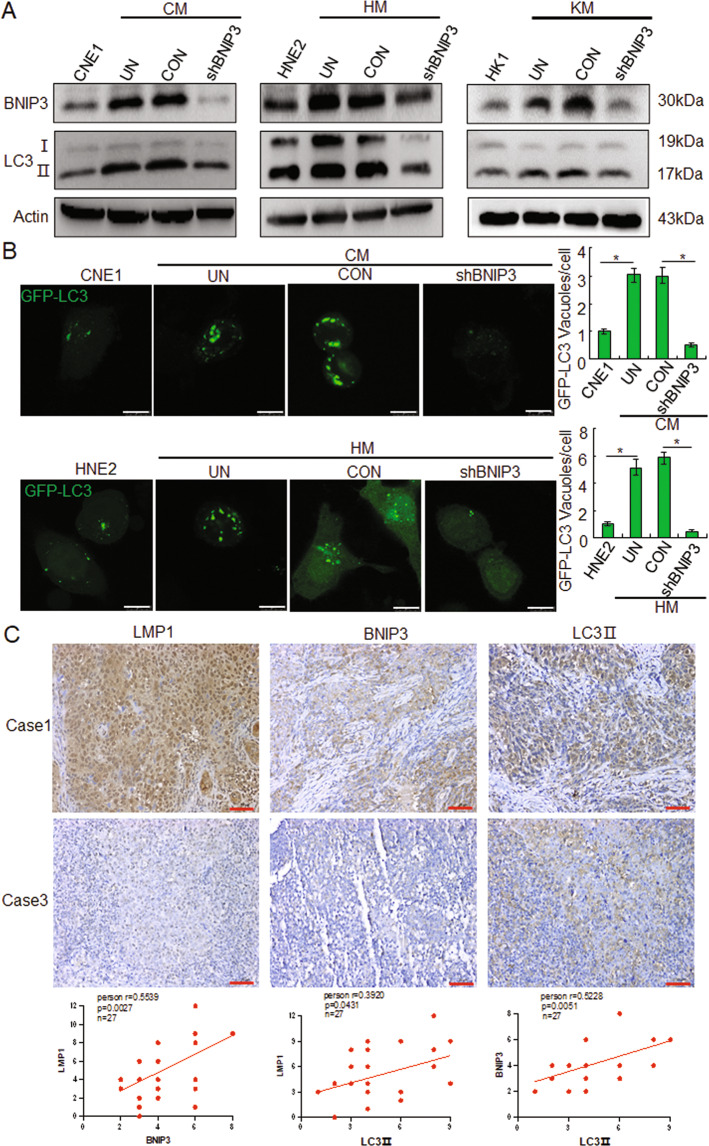
LMP1 induces autophagy through BNIP3 in NPC cells. **A** After transfection with shBNIP3 and control for 24 h, the expression of BNIP3 and LC3І/ІІ in CNE1, CM, HNE2, HM, HK1, and KM cells by Western blots. **B** After transfection with shBNIP3 and control for 24 h, accumulation of GFP-LC3 puncta in NPC cells, Scale bar: 10 μm. Data are expressed as means ± S.D. of three experiments. *p < 0.05. **C** Immunohistochemical analysis was performed to detect the expression of LMP1, BNIP3, and LC3II in NPC patient tissues, and the correlations between LMP1, BNIP3, and LC3II were analyzed. UN untreated, CON negative control, Scale bar: 50 μm.

In order to determine whether the BNIP3 involved in LMP1 mediated autophagy, targeting BNIP3 shRNA were transfected into CM, HM, and KM cells. The data showed that knockdown of BNIP3 could significantly reduce the LMP1 mediated LC3II expression (Figs. 3A and S1). Immunofluorescence experiment results also indicated that knockdown of BNIP3 could obviously suppress the LC3 puncta, which was induced by the EBV-LMP1 (Fig. 3B). Furthermore, IHC staining of 27 clinical patient samples indicated the expression of BNIP3 and LC3 II in LMP1 positive tissues (Fig. 3C). Spearman correlation analysis between LMP1, BNIP3, and LC3 II was confirmed based on the IHC staining score. The results showed that LMP1 and BNIP3 (*r* = 0.5539, *p* = 0.0027), LMP1 and LC3 II (*r* = 0.0.3920, *p* = 0.0431), and BNIP3 and LC3 II (*r* = 0.5228, *p* = 0.0051) had a positive correlation. These findings suggested that LMP1 induces autophagy through BNIP3 in NPC cells.

### LMP1 increases radioresistance of NPC cells through BNIP3

**Fig. 4:**
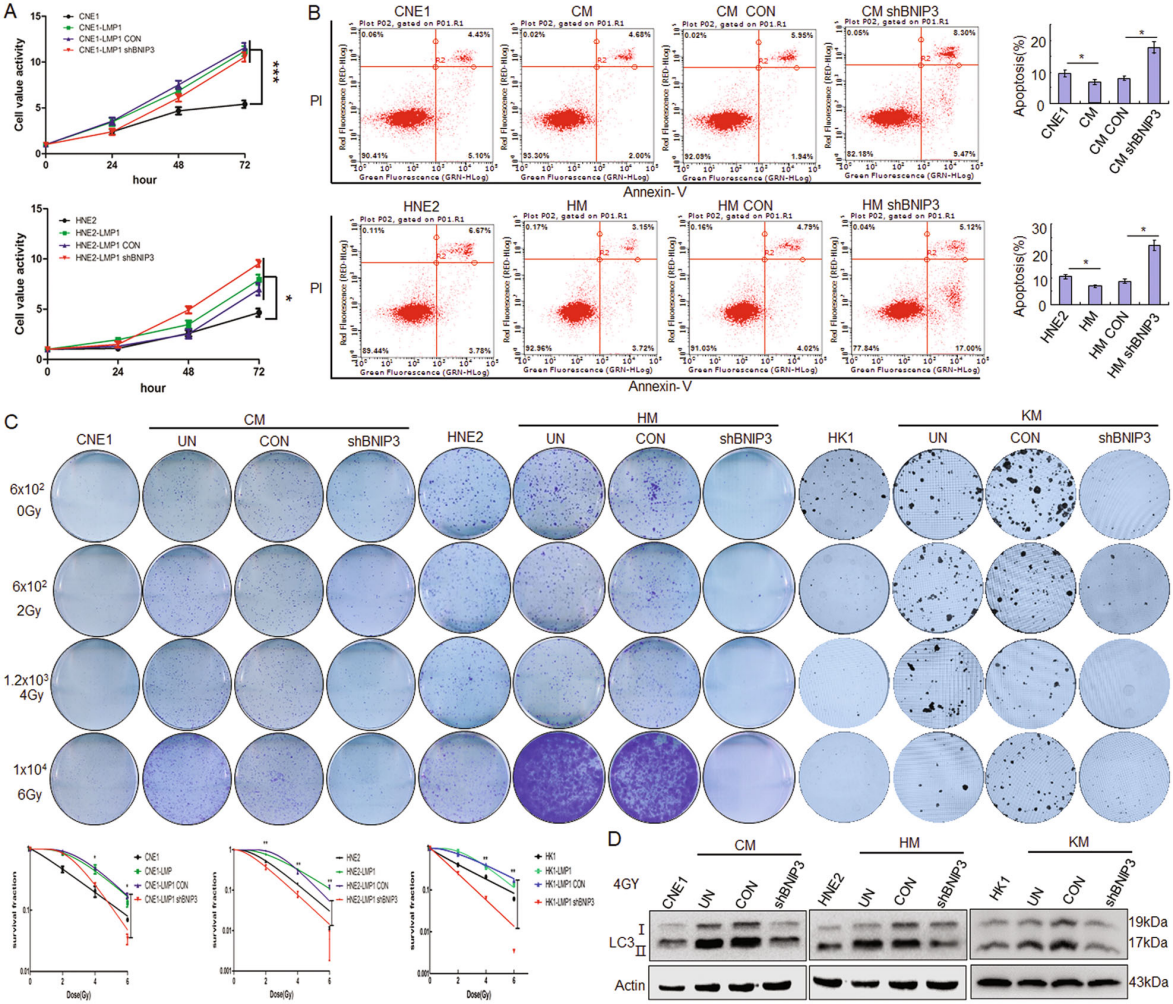
LMP1 increases radioresistance of NPC cells through BNIP3. **A** After transfection with shBNIP3 and control for 24, 48, and 72 h, respectively, the proliferation of different NPC cells was detected by MTS assay. **B** After transfection with shBNIP3 and control for 24 h, flow cytometric analysis was performed to observe cell apoptosis. **C** After transfection with shBNIP3 and control for 24 h, CM, HM, and KM cells were treated with IR at 0, 2, 4, and 6 Gy and cultured for 2 weeks. Then, fixation, staining, and colony quantification were gradually executed. Top: the representative images of hexamethylpararosaniline stained clonies. Bottom: the survival curves of CM, HM, and KM cells were fit to the data using a linear quadratic model of radiation sensitivity. Data are expressed as means ± S.D. of three experiments. *p < 0.05, **p < 0.01. **D** After transfection with GFP-LC3 for 24 h and with IR at 4 Gy, the expression of LC3 I/II in CM, HM, and KM cells by Western blots. UN untreated, CON negative control.

The function of BNIP3 has been associated with both cell death and cell survival^16^. To determine the influence of BNIP3 mediated by LMP1 on NPC cell proliferation, the MTS results showed that the proliferation ability of LMP1-positive NPC cells was stronger than that of LMP1-negative NPC cells, but the proliferation ability of CM and HM cells with knockdown of BNIP3 showed no obvious change compared to the control group (Fig. 4A). Further, the flow cytometry results showed that the apoptosis of LMP1-positive NPC cells was lower than that of LMP1-negative NPC cells, and there was increased apoptosis of CM and HM after knockdown of BNIP3 compared to the control group (*p* < 0.05) (Fig. 4B). These results show that LMP1-induced BNIP3 can inhibit apoptosis and have no obvious effect of proliferation in NPC cells. Clinically, the radioresistance of NPC is the key factor affecting the therapeutic effect^17^, and autophagy is an important mechanism to regulate radioresistance^12^. Therefore, we want to know whether BNIP3 and its induced autophagy are involved in the regulation of radioresistance. The colony formation assays results showed that colony formation of LMP1-negative NPC cells, whether treated or not treated with irradiation, was weaker than that of LMP1-positive NPC cells (*p* < 0.01), which indicated that LMP1 could increase cellular radioresistance. After knockdown of BNIP3, colony formation of LMP1-positive CM, HM, and KM cells were significantly decreased compared to control groups (*p* < 0.01) (Fig. 4C). Meanwhile, Western blot results also showed that knockdown of BNIP3 could significantly inhibit LC3II expression (Fig. 4D). These results suggest that BNIP3 could increase the radioresistance through increased autophagy in NPCs.

### EBV-LMP1 induces BNIP3 through the ERK/HIF1α pathway

**Fig. 5:**
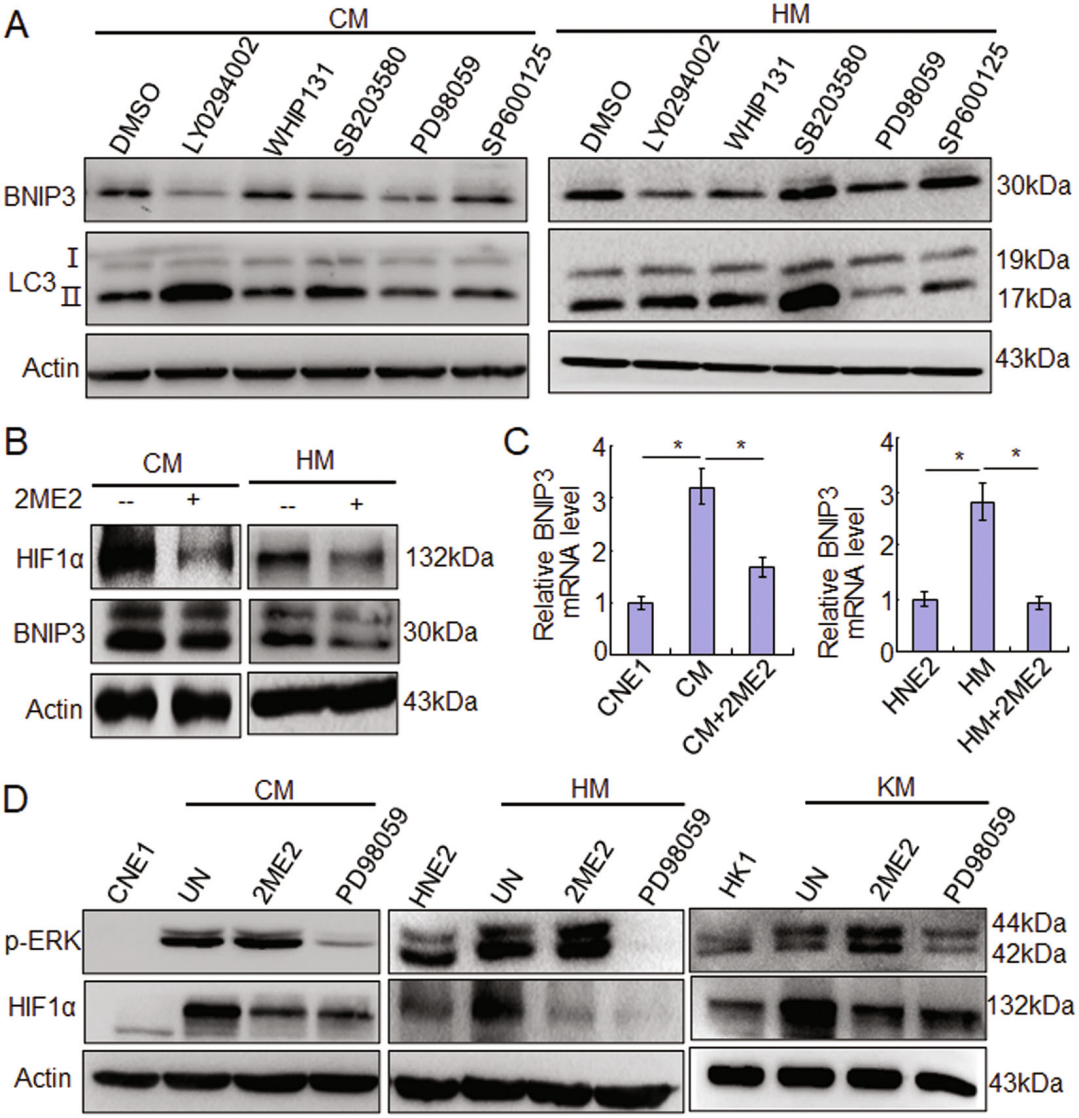
EBV-LMP1 induces BNIP3 through the ERK/HIF1α pathway. **A** After treatment of different inhibitors for 24 h, the expression of BNIP3 and LC3І/ІІ in NPC cells by Western blots. **B** After treatment of 2ME2 for 24 h, the expression of HIF1α and BNIP3 in NPC cells by Western blots. **C** After treatment of 2ME2 for 24 h, the mRNA expression of BNIP3 in NPC cells by quantitative RT-PCR. Data are expressed as means ± S.D. of three experiments. *p < 0.05. **D** After treatment of 2ME2 or PD98059 for 24 h, the expression of p-ERK and HIF1α in CM, HM, and KM cells by Western blots. UN untreated.

Some studies have reported that LMP1 triggers several signal pathways, such as MAPK, JAK/STAT, PI3K/AKT, NF-κB, and others, to regulate multiple functions in NPC cells^4,5^. To determine which pathway is mainly responsible for inducing BNIP3 expression and autophagy, the inhibitors targeting PI3K/AKT (LY294002), JAK3/STAT (WHIP131), p38 (SB203580), ERK (PD98059), and JNKs (SP600125) were used. The results showed that in CM cells, BNIP3 expression decreased after treatment with LY294002 and PD98059. Importantly, LC3II expression decreased only after treatment with PD98059. In HM cells, the BNIP3 expression decreased after treatment with LY294002, WHIP131, and PD98059, and the LC3II expression also decreased only after treatment with PD98059 (Figs. 5A and S2). These results indicate that the ERK signaling pathway is mainly responsible for BNIP3 upregulation and autophagy induced by LMP1 in NPC cells. Furthermore, HIF1α is a key molecule for regulating BNIP3^18–20^. Therefore, we analyzed whether HIF1α is involved in regulation of LMP1 on BNIP3in LMP1-positive NPC cells with HIF1α inhibitor 2ME2 treatment, and the data showed that with the inhibition of HIF1α, BNIP3 mRNA and protein expression decreased accordingly (Fig. 5B, C). Moreover, the results indicated that HIF1α decreased, but the p-ERK expression did not change after 2ME2 treatment, while both the p-ERK and HIF1α decreased after PD98059 treatment in CM, HM and KM cells (Fig. 5D). These results suggest the ERK-HIF1α signal axis is responsible for LMP1 induced BNIP3 expression and autophagy in NPC cells.

### LMP1-induced BNIP3 triggers autophagy through a dissociated Bcl-2-Beclin1 complex

**Fig. 6:**
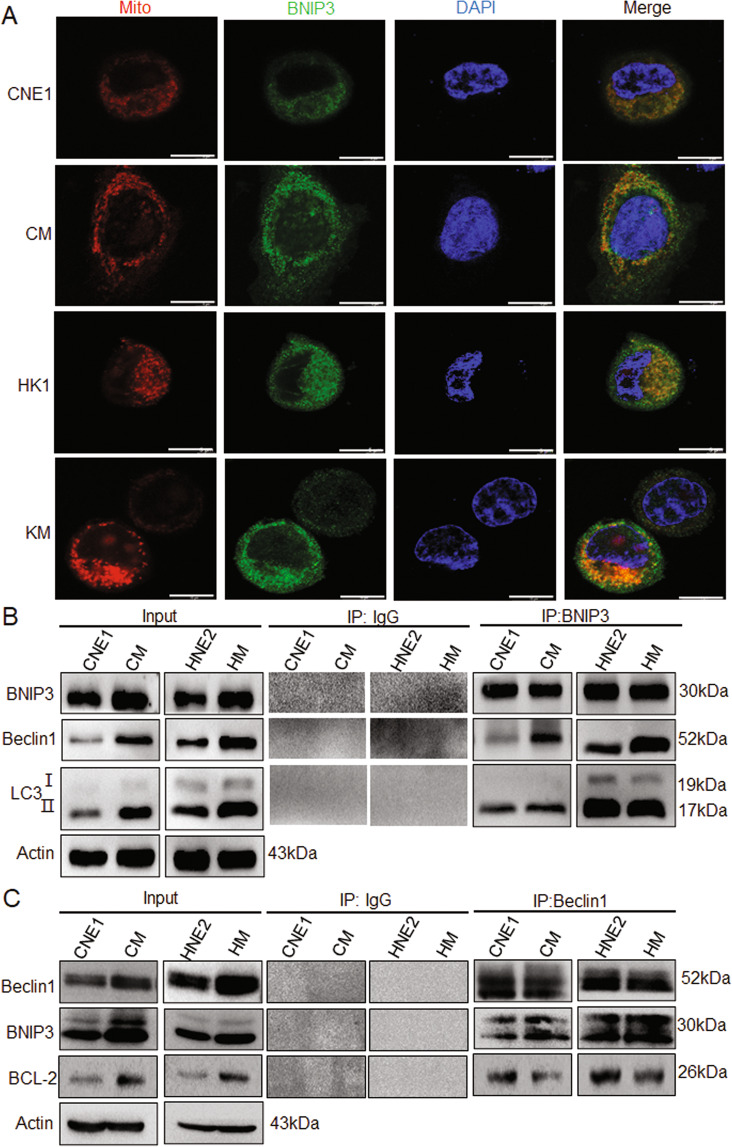
LMP1-induced BNIP3 triggers autophagy through a dissociated Bcl-2-Beclin1 complex. **A** After MitoTracker Red staining, the colocalization of BNIP3 in NPC cells CNE1, CM, HK1, and KM was detected by immunofluorescence assay. Scale bar: 10 μm. **B** The interaction of BNIP3 with Beclin1 and LC3II was detected by Co-IP. **C** The interaction of Beclin1 with BNIP3 and Bcl-2 was detected by Co-IP.

It has been proven that the role of BNIP3 in autophagy has three main forms: the first is that under the condition of hypoxia, BNIP3 forms dimer, which locates on the outer membrane of the mitochondria and causes mitochondrial damage, inducing autophagic cell death. The second is that BNIP3 dimer locates on the mitochondria and interacts with the autophagosome-localized LC3, and causes mitophagy. The third is that BNIP3 can destroy the structure of Bcl-2 and Beclin1 complex and release Beclin1 through the competitive binding with Bcl-2, thus promoting autophagy^21^. To clarify which mechanism is involved in LMP1-induced protective autophagy, the location of BNIP3 and the relationship among BNIP3, Beclin1, LC3, Bcl-2, and autophagy were investigated. It was found that BNIP3 was located on the mitochondrial membrane and LMP1 promoted BNIP3 expression in NPC cells by immunofluorescence analysis in CM and KM cells (Fig. 6A). Further, the immunoprecipitation analysis showed that LMP1 increased the Beclin1, but not LC3, to bind with BNIP3 (Fig. 6B). Furthermore, we found that LMP1 dramatically decreased the binding between Bcl-2 and Beclin1 compared to the control, through immunoprecipitation. However, only part of the Beclin1 was displaced with BNIP3, so the Beclin1-Bcl-2 complex was still detectable at a reduced level in the LMP1-positive NPC cells (Fig. 6C). Taken together, these results suggest that LMP1-induced BNIP3 expression triggers protective autophagy through increasing the disruption of the Bcl-2-Beclin1 complex.

### LMP1-induced BNIP3 increases radioresistance of NPC in vivo

**Fig. 7:**
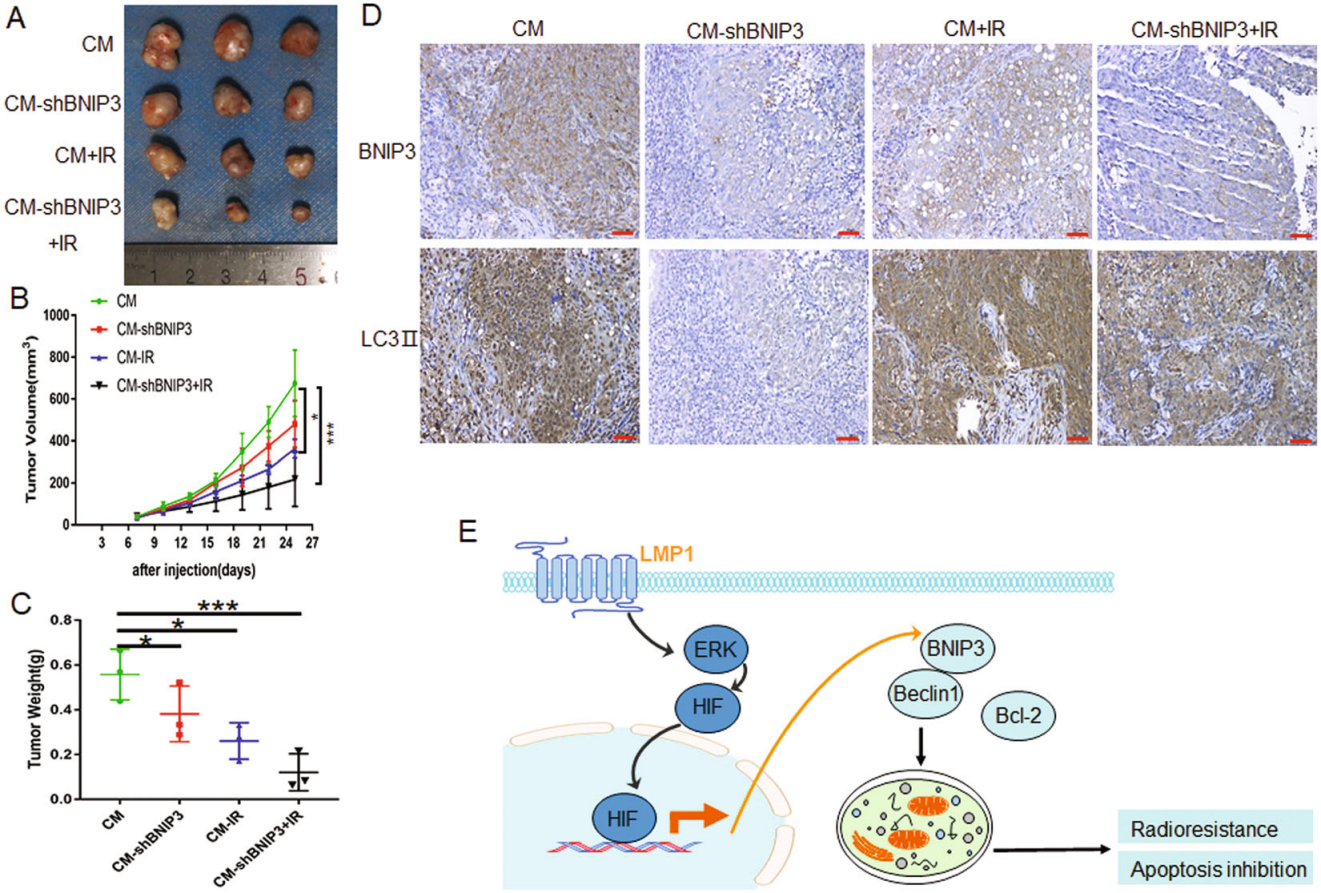
LMP1-induced BNIP3 increases radioresistance of NPC in vivo. The xenograft model was established using CM cells or CM-shBNIP3 cells. When the tumor volume reached 60–100 mm^3^, 6 Gy local irradiation was administered. **A** At the experimental end point, tumor xenografts were dissected and photographed. **B** Tumor volume was measured every 3 days after injection. **C** Tumor weight was measured at the experimental end point. *p < 0.05, ***p < 0.001. **D** Representative immunohistochemistry (IHC) staining of BNIP3 and LC3II expression in CM xenografts in the groups indicated in **A**. Scale bar: 50 μm. **E** A schematic illustration of the LMP1 promoting radioresistance by inducing autophagy through ERK/HIF1α/BNIP3 in NPC.

To investigate whether LMP1-induced BNIP3 increases radioresistance through protective autophagy in vivo, animal experiments were performed. As shown in Fig. 7A–C, although knockdown of BNIP3 alone or radiation treatment could inhibit tumor growth compared with control groups (*p* < 0.05), the combined effect of BNIP3 and radiation significantly reduced the tumor growth (*p* < 0.001). Further, IHC staining of the tumor xenograft tissue showed that knockdown of BNIP3 could effectively reduce the expression of LC3II, although radiation could promote the expression of LC3 to a certain extent (Fig. 7D). These data demonstrated that LMP1-induced BNIP3 could increase the radioresistance through protective autophagy in vivo.

We thus propose a simple model (Fig. 7E) in which the LMP1-induced BNIP3 through ERK/HIF1α are crucial to the disruption of an interaction between Bcl-2 and Beclin1, thereby releasing the negative autophagic blockade, and further triggering the radioresistance of the NPC tumor.

## Discussion

Autophagy has been described as a form of cell death for a variety of cell types. However, in the context of hypoxia, nutrient depletion, or growth factor deprivation, it is clear that autophagy is crucial in maintaining cellular ATP production and macromolecular synthesis and, therefore, represents an essential pro-survival pathway^10^. Therefore, there are two opposing views about the relationship between autophagy and radiotherapy resistance. Some researchers think that autophagy can promote radiosensitivity, but others disagree. For example, Kuwahara et al. reported that an autophagy inhibitor, 3-methyladenine, induced radioresistance of liver cancer cells^22^. Liang’s group found that induction of autophagy with rapamycin promoted glioma-initiating cell sensitivity to irradiation^23^. Conversely, Chaachouay et al. found that autophagy contributes to resistance of breast cancer cells to ionizing radiation^24^. Ko et al. also found that autophagy can inhibit radiosensitization in vitro, yet reduces radioresponses in vivo due to deficient immunogenic signaling^25^. In our present study, the results showed that EBV-LMP1 was involved in the radioresistance of NPC cells by promoting cell protective autophagy.

The BH3-only family member BNIP3 has been described as pro-death, as well as having pro-autophagic and protective functions, depending on the type of stress and cellular context^16^. Most studies have reported that BNIP3 can induce apoptosis. For example, Verticillin A induced BNIP3 expression in human colon carcinoma, and hepatoma has been reported to correlate with apoptosis^26^. Conversely, there are still some studies suggesting that BNIP3 can inhibit the apoptosis of tumor cells^27,28^. On the other hand, BNIP3 has been reported to induce autophagy in tumor cells. However, whether this leads to cell death or survival is controversial, as the induction of autophagy by BNIP3 is associated with autophagic cell death, but it has been found to have a protective effect in some conditions. Chourasia et al. reported that mitophagy defects arising from BNIP3 loss promote mammary tumor progression to metastasis^29^. Wu et al. found that BNIP3 can inhibit the growth of tumor cells by regulating autophagy in the process of Ras-regulated tumor growth^30^. In contrast, Nollet et al. found that the integrins α6β1 and Bnip3 promote survival of CRPC cells selectively on laminin, through the induction of autophagy and mitophagy^31^. Li et al. also found that p53 repressed BNIP3 transcription and activity, leading to mitophagy arrest. The defective mitophagy impaired mitochondrial homeostasis, evoked cellular oxidative stress, and initiated mitochondrial apoptosis^32^. These studies fully illustrate the diversity of action modes and biological functions of BNIP3 under different conditions.

In our present study, although knockdown of BNIP3 had no significant effect on NPC cell survival, the significant enhancement of radiosensitivity following BNIP3 knockdown indicates that BNIP3-induced autophagy is indispensable for cell survival under IR treatment. In addition to autophagy, BNIP3 inhibition also leads to the appearance of apoptosis. It may be that defective mitophagy impairs mitochondrial homeostasis, therefore initiating apoptosis. For NPC, LMP1 mediated BNIP3-induced autophagy appears to be a protective process, probably acting as a warning signal for cells to anticipate irradiation stress.

At present, many signaling pathways have been demonstrated as being involved in regulating BNIP3. Sun et al. found that the activation of the ERK/HIF-1 signaling pathway in liver cancer cells can promote BNIP3 dependent autophagy, and participate in cell apoptosis resistance^19^. Riis et al. confirmed that IGF-1 induced BNIP3 expression through AKT1-mediated inhibitory phosphorylation of GSK-3β^33^. We also observed that the increased autophagy in the LMP1-positive cells is mainly mediated through the extracellular ERK/HIF1α signaling axis. It has been shown that there are several ways BNIP3 mediates autophagy; the first is that under hypoxia, BNIP3 causes mitochondrial injury induced autophagic cell death^21^. The second is that BNIP3 dimers locate the mitochondria and interact with the autophagosome-localized LC3, causing mitophagy^34,35^. The third is that BNIP3 can destroy the structure of Bcl-2 and Beclin1 complexes and release Beclin1, thus promoting autophagy^21,31^. In this report, we demonstrated that LMP1-induced autophagy is controlled by BNIP3. In particular, BNIP3 triggers autophagy through dissociated Bcl-2-Beclin1 complexes, and this protective autophagy might be required for the protection of NPC cells from irradiation-induced cell death.

In previous studies, we demonstrated that LMP1 plays an important role in the radioresistance of NPC cells. LMP1 represses the DNA damage response (DDR) through DNA-dependent protein kinase DNA-PK/AMPK signaling, and then promotes resistance to apoptosis induced by irradiation^6^. Meanwhile, LMP1 promotes hexokinase 2 (HK2) through c-Myc, and the upregulation of HK2 elevated aerobic glycolysis and facilitated proliferation by blocking apoptosis and especially cause resistance of NPC cells to radiation therapy, leading to the poor overall survival of NPC patients^7^. On the other hand, EBV-LMP1 promote VEGF expression and angiogenesis through the JNKs/c-Jun signaling pathway, and LMP1-targeted DNAzyme increases the radiosensitivity of NPC by inhibiting HIF-1/VEGF activity. These studies analyzed the molecular mechanism of EBV-LMP1 involved in radioresistance of NPC cells from the DNA damage repair, metabolism, and angiogenesis^8^. In present study, we further explored the protective autophagy mediated by LMP1 through ERK/HIF1α/BNIP3 pathway to promote the radioresistance of tumor cells. These studies fully show that LMP1 can play an important role in NPC radioresistance through a variety of mechanisms, and our current work expands the understanding of the molecular mechanism of LMP1 in the NPC development.

## Materials and methods

### Cell cultures, transfection and reagents

CNE1, HNE2 and HK1 are LMP1 negative NPC cell lines. CNE1-LMP1(CM), HNE2-LMP1(HM) and HK1-LMP1(KM) are cell lines that are stably transfected with EBV-LMP1^6,7^. The source of these cell line had been identified and authenticated, and the mycoplasma contamination has been tested. The cells were cultured in RPMI-1640 medium supplemented with 10% fetal bovine serum (FBS, Gibco BRL, Gaithersburg, MD, USA), 100 IU/mL penicillin, 100 mg/mL streptomycin, and 2 mM/L l-glutamine in a humidified atmosphere of 5% CO_2_ at 37 °C. The cell transfection used Lipofectamine 2000 (Invitrogen, Carlsbad, CA, USA) according to the manufacturer’s instructions. The pGV141-LMP1-wt were purchased from Genechem (Shanghai, China). The shRNA targeting BNIP3 and control-shRNA were synthesized by Ribo Bio-Technology (Guangzhou, China), and the sequences of shRNA are described in Table S1. The AKT inhibitor (LY294002), JAK3 inhibitor (WHIP131), p38 inhibitor (SB203580), MEK inhibitor (PD98059), and JNKs inhibitor (SP600125) were purchased from Selleck Chemicals (Houston, TX, USA). The HIF1α inhibitor (2ME2) was obtained from Cayman Chemical (Ann Arbor, MI, USA), and MitoTracker™ Red was purchased from Invitrogen (M7512).

### Transmission electron microscopy

The NPC cells were harvested by trypsin digestion and fixed with 2.5% glutaraldehyde on ice for 2 h, followed by postfixation in 2% osmiumtetroxide. Then, the cells were immersed in SPIPON812 resin after dehydrating with sequential washes in 50, 70, 90, 95, and 100% acetone. The ultrathin sections (50–100 nm) were collected on copper grids and counterstained using 3% uranyl acetate and leadcitrate. Images were taken with a Tecnai G2 Spirit TEM (FEI, Thermo Fisher Scientific, Waltham, MA, USA).

### Immunofluorescence

The cells were transfected with pEGFP-LC3 plasmid (Addgene, plasmid#: 24920). For fluorescence analysis, cell samples were visualized with confocal microscopy (LSM 510 META, Carl Zeiss, Germany). The average value is the number of puncta in at least 50 randomly selected individual cells under each condition.

### Quantitative RT-PCR

Total RNA was isolated from cells with the TRIzol reagent (15596026, Invitrogen) and reverse transcribed into cDNA (K1621, Thermo Fisher Scientific), according to the manufacturer’s instructions. Quantitative PCR was performed using an ABI 7500 instrument (Foster City, CA, USA) with TaqManTM Gene Expression Master Mix (4369016, Thermo Fisher Scientific). For the qPCR array, the selected 88 autophage-associated genes were mainly referred to the gene information from the commercial chip such as PAHS-084Z autophagy gene chip (SABioscience, Frederick, MD, USA), and autophage-associated genes reported in the literatures. β-Actin was used as an internal control. The primers are listed in Table S2.

### Western blotting

Cells were lysed with IP lysis buffer containing a 10% cocktail (B14001, Bimake, Houston, TX, USA), and the protein concentration was determined using a BCA kit (Pierce Chemical, Rockford, IL, USA). SDS-PAGE was performed using 30–50 μg of total cell protein. Then, the protein was transferred to a PVDF membrane, blocked with 5% skim milk at room temperature, and incubated with the indicated antibodies. The primary antibodies used were anti-LMP1 (M0897, 1:500) purchased from DAKO (Glostrup, Denmark), and anti-LC3 obtained from NOVUS (NB100-2220, 1:500, Littleton, CO, USA). Anti-BNIP3 (ab10433, 1:1000) and anti-Beclin1 (ab207612, 1:1000) were purchased from Abcam (Cambridge, MA, USA). Anti-p-Akt (ser473, 9270, 1:1000), anti-p-ERK1/2 (4370, 1:1000), anti-p-JNK (Thr183/Tyr185, 9251, 1:1000), anti-p-JAK3 (Tyr980/981, 5031, 1:1000), anti-rabbit IgG-HRP (14708, 1:2000), and anti-mouse IgG-HRP (14709, 1:2000) antibodies were obtained from cell signaling technology (Danvers, MA, USA). Anti-Bcl-2 (sc-7382, 1:500), anti-p-P38 (thr180/try182, sc-4511, 1:1000), anti-HIF1α (sc-53546, 1:500), and anti-β-actin (sc-8432, 1:2000) antibodies were obtained from Santa Cruz Biotechnology (Dallas, Texas, USA). Blots were analyzed using a chemiluminescence imaging system (Bio-Rad, Hercules, CA, USA).

### Co-immunoprecipitation analysis (Co-IP)

For the Co-IP, cell lysates were clarified by immunomagnetic separation and incubated with the indicated antibody plus Dynabeads® Protein A (Thermo Scientific, 10002D) at 4 °C overnight to form immunocomplexes. After extensive washing with lysis buffer, the immunocomplexes were analyzed by western blotting, as described above.

### Cell viability assay

Cell viability was measured by MTS kit (G5421, Promega, Madison, WI, USA) following the manufacturer’s instruction. Cells were cultured in a 96-well plate with differently treatments for the indicated time, and then the assay solution was added for an incubation of 1.5 h. The samples were measured by a Microplate Reader (BioTek ELx800, Winooski, VT, USA) at 490 nm.

### Flow cytometry analysis

1–5 × 10^5^ cells from each sample were digested with 0.25% trypsin and fixed in 70% cold ethanol overnight after resuspending in D-hanks three times. The cells were subsequently collected and were treated according to the instructions of the Annexin-V FITC/PI Apoptosis Detection Kit (KGA107, KeyGEN BioTECH, Jiangsu, China). All samples were analyzed using flow cytometry (MoFlo XDP, Beckman Coulter, Miami, FL, USA) according to the manufacturer’s protocols.

### X-ray irradiation

The cells and mice irradiation experiments were carried out on a PXI X-RAD 225 system (Precision X-ray Inc., North Branford, CT) at indicated dosages.

### Colony formation assay

The radiosensitivity was measured by colony-formation assay as previously described^36^. Briefly, a gradient number of NPC cells with different treatments were seeded into 6-well dishes and cultured for another 24 h. After the cells were attached to the plates, the cells were irradiated with X-rays (0, 2, 4, or 6 Gy), then were further cultured for 2 weeks to allow colony formation. Colonies in dishes were stained with 0.0125% crystal violet (Sigma-Aldrich, St. Louis, MO, USA), and counted for the number of surviving colonies (defined as a colony with >50 cells). The data were analyzed using the linear-quadratic model, and the surviving fraction was calculated as the ratio of the plating efficiency of the treated cells compared to control cells using GraphPad Prism (GraphPad Software, La Jolla, CA, USA).

### Animal experiments

Animal experiments were conducted with the approval of the Institutional Animal Care and Use Committee of the Xiangya School of Medicine of Central South University and conform to the legal mandates and federal guidelines for the care and maintenance of laboratory animals. Here, no blinding was done in animal experiments. Twelve six-week-old female athymic nude mice (BALB/C) were injected with 5 × 10^6^ CM cells or 5 × 10^6^ CM-shBNIP3 cells resuspended in 100 μL PBS. Tumor volumes were calculated using the formula: (length $*$ width^2^) $*$ (*π*/6). The animals were randomly divided into control group and the irradiation treatment (IR) group (*n* = 3 per group) when the tumor volume reached 60–100 mm^3^, and 6 Gy local irradiation was administered in groups of mice subjected to IR. After 3 weeks, the mice were killed, and the tumors were collected and fixed with 10% buffered formalin for IHC analysis.

### Immunohistochemistry (IHC)

A total of 27 Paraffin-embedded tumor tissue samples with clinical details of NPC patients (from 2017 to 2019) were collected from the Pathology Department of Xiangya Hospital (Table S3). IHC was performed using a Histomouse SP Broad Spectrum DAB kit (Invitrogen-Zymed, Carlsbad, CA, USA) following the manufacturer’s instructions. The stained sections were independently examined by two of the authors (Z.Z. and J.P.). A semi-quantitative evaluation of the positivity of each protein by IHC was performed using a method described previously^8^.

### Statistical analysis

Statistical analysis was performed with the SPSS statistical software program (ver.19.0). Data are presented as means ± SD. Differences between various groups were evaluated using a two-tailed Student’s *t*-test and a *p* value < 0.05 was considered statistically significant.

## Supplementary information

Supplementary Information
