# Supplementary material for: EBV-LMP1 promotes radioresistance by inducing protective autophagy through BNIP3 in nasopharyngeal carcinoma
Source: Cell Death Dis. 2021 Apr 1;12(4):344. doi: 10.1038/s41419-021-03639-2 (PMC8016912; doi:10.1038/s41419-021-03639-2)
Supplement: Supplementary file 1 — Supplementary Information [file 41419_2021_3639_MOESM1_ESM.pdf]

Supplementary Figures

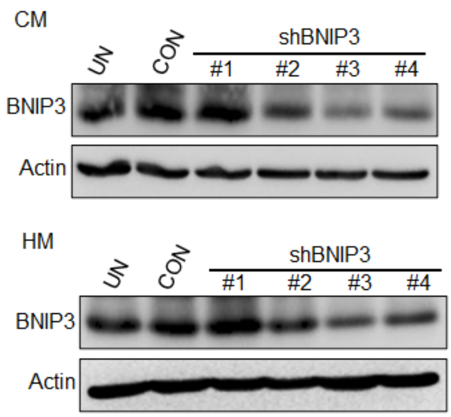

Figure S1. Analysis of the effect of targeting BNIP3 shRNA. CM and HM cells were respectively transfected with four BNIP3 shRNAs, and the expression of BNIP3 was analyzed by western blot.

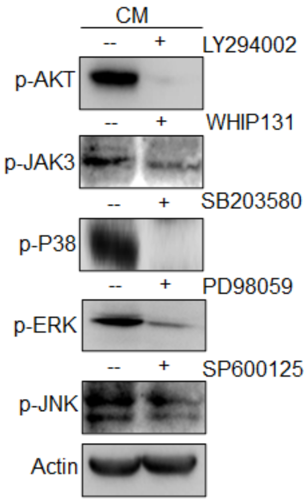

Figure S2. Effect analysis of various kinase inhibitors in CM cells. CM cells were treated with the indicated concentrations of LY294002, WHIP131, SB203580, PD98059, or SP600125 for 24 h, western blot was used to determine the effect of the inhibitors.

Supplementary tables

Table S1. shBNIP3 target sequence for RNA interference

ID	target sequence (5' - 3')	start position
BNIP3-RNAi (34321)	*TCTCTCATTTGCTGGCCAT*	*636*
BNIP3-RNAi (34322)	*CTCGGTTTCTATTTATAAT*	*226*
BNIP3-RNAi (34323)	*ACTGCACTTCAGCAATAAT*	*184*
BNIP3-RNAi (34324)	*CACGTCACTTGTGTTTATT*	*800*
Control	*TTCTCCGAACGTGTCACGT*	

Table S2. Primers for quantitative RT-PCR

**Gene name**	**Forward sequence (5' to 3')**	**Reverse sequence (5' to 3')**
AKT1	AGCGACGTGGCTATTGTGAAG	GCCATCATTCTTGAGGAGGAAGT
ALPS1A	AGATTGTGTGATGAAGGACATGG	TGTTGCTGGTGAGTGTGCATT
ALPS1B	TGCCTTGGTAGGATTGGGC	GCTGGTAGACTCTCGGAGTTC
AMBRA1	TGGGGAGGTTAGGATTTGGGA	GAGCCGTAGGGTGGAAAGC
AMPK	TTGAAACCTGAAAATGTCCTGCT	GGTGAGCCACAACTTGTTCTT
APP	TCTCGTTCCTGACAAGTGCAA	GCAAGTTGGTACTCTTCTCACTG
Atg10	AGACCATCAAAGGACTGTTCTGA	GGGTAGATGCTCCTAGATGTGAC
Atg12	CTGCTGGCGACACCAAGAAA	CGTGTTCGCTCTACTGCCC
Atg13	TTGCTATAACTAGGGTGACACCA	CCCAACACGAACTGTCTGGA
Atg14	GCGCCAAATGCGTTCAGAG	AGTCGGCTTAACCTTTCCTTCT
Atg16L	AACGCTGTGCAGTTCAGTCC	AGCTGCTAAGAGGTAAGATCCA
Atg16L2	TGGACAAGTTCTCAAAGAAGCTG	CCTCAGTGCGACCAGTGAT
Atg17	ATCGAAGAGTGTGTACCTACAGT	GCAGGTGGACGATCACATAAGAT
Atg2A	GCTCAGGGTACATGGAGCTG	CTCGTGGTCTGTAAGGCTCAC
Atg2B	AACTGCTGACGAATCCTCAGG	GGGGTTCCAGCTAGGTGAGA
Atg3	GACCCCGGTCCTCAAGGAA	TGTAGCCCATTGCCATGTTGG
Atg4A	TGCTGGTTGGGGATGTATGC	GCGTTGGTATTCTTTGGGTTGT
Atg4B	AGTTGGCGAAGGCAAGTCC	CCACGTATCGAAGACAGCAAG
Atg4C	GCTTTGACAACAGACTGTGGG	TGTAGTATGAGTCCTTGAGCCAA
Atg4D	CCAGCCCACTGTGGATGTC	AAGCCCACGGTACAGCTTG
Atg5	AGAAGCTGTTTCGTCCTGTGG	AGGTGTTTCCAACATTGGCTC
Atg7	ATGATCCCTGTAACTTAGCCCA	CACGGAAGCAAACAACTTCAAC
Atg9A	CTGCCCTTCCGTATTGCAC	CTCACGTTTGTGGATGCAGAT
Atg9B	CTGGGGCCGAGTACAACAAG	CTGGGCAATTCGGGAAATGGA
BAK1	ATGGTCACCTTACCTCTGCAA	TCATAGCGTCGGTTGATGTCG
BAX	CCCGAGAGGTCTTTTTCCGAG	CCAGCCCATGATGGTTCTGAT
BCL2	GGTGGGGTCATGTGTGTGG	CGGTTCAGGTACTCAGTCATCC
BCL2L1	GAGCTGGTGGTTGACTTTCTC	TCCATCTCCGATTCAGTCCCT
BCL2L8	CCCAGAGTTTGAGCCGAGTG	CCCATCCCTTCGTCGTCCT

BECN1	CCATGCAGGTGAGCTTCGT	GAATCTGCGAGAGACACCATC
BID	ATGGACCGTAGCATCCCTCC	GTAGGTGCGTAGGTTCTGGT
BNIP3	CAGGGCTCCTGGGTAGAACT	CTACTCCGTCCAGACTCATGC
CASP3	CATGGAAGCGAATCAATGGACT	CTGTACCAGACCGAGATGTCA
CASP8	TTTCTGCCTACAGGGTCATGC	GCTGCTTCTCTCTTTGCTGAA
CDKN1B	AACGTGCGAGTGTCTAACGG	CCCTCTAGGGGTTTGTGATTCT
CLN3	CGCCCACGACATCCTTAGC	AGCAGCCGTAGAGACAGAGTT
CTSB	GAGCTGGTCAACTATGTCAACA	GCTCATGTCCACGTTGTAGAAGT
CXCR4	ACTACACCGAGGAAATGGGCT	CCCACAATGCCAGTTAAGAAGA
DAPK1	ACGTGGATGATTACTACGACACC	TGCTTTTCTCACGGCATTTCT
DRAM	CGTCAGCCGCCTTCATTATCT	TCCAAGCACTAAAGACACCAAG
DRAM2	CTGTGCTTACCTTTGGTATGGG	GCACTTACTCCACACCAGATAAC
EIF2AK3	ACGATGAGACAGAGTTGCGAC	ATCCAAGGCAGCAATTCTCCC
EIF4G1	CCCGAAAAGAACCACGCAAG	TTCCCCTCGATCCTTATCAGC
ESR1	GAAAGGTGGGATACGAAAAGACC	GCTGTTCTTCTTAGAGCGTTTGA
FADD	GCTGGCTCGTCAGCTCAAA	ACTGTTGCGTTCTCCTTCTCT
GABARAP	ACTCGCTGGAACACAGATGC	TCTGAGAGCCTGAGACCTTTT
GABARAPL1	ATGAAGTTCCAGTACAAGGAGGA	GCTTTTGGAGCCTTCTCTACAAT
GABARAPL2	ACTCGCTGGAACACAGATGC	TCTGAGAGCCTGAGACCTTTT
HDAC1	CCGCATGACTCATAATTTGCTG	ATTGGCTTTGTGAGGGCGATA
HDAC6	GAGGGAGAACTCCGTGTCCTA	AATAGCCATCCATAAGACTGTGC
HMGB1	TATGGCAAAAGCGGACAAGG	CTTCGCAACATCACCAATGGA
HSP90	CCGGTGTAGGAATGACCAGAG	TTAAAAACTCGCTTGTCCCAGAT
HSP90A	AGGAGGTTGAGACGTTCGC	AGAGTTCGATCTTGTTTGTTCGG
HSPA8	ACTCCAAGCTATGTCGCCTTT	TGGCATCAAAAACTGTGTTGGT
HTT	AGCTCACCGCTGCTAAGGA	ACATCCGATCTCGATTCAGAGTC
IFNG	TCGGTAACTGACTTGAATGTCCA	TCGCTTCCCTGTTTTAGCTGC
IGF1	GCTCTTCAGTTCGTGTGTGGA	GCCTCCTTAGATCACAGCTCC
INS	GCAGCCTTTGTGAACCAACAC	CCCCGCACACTAGGTAGAGA
IRGM	GCCATGAATGTTGAGAAAGCCT	GTCCTGGACACTATCTTCAGAGT
LAMP1	TCTCAGTGAACTACGACACCA	AGTGTATGTCCTCTTCCAAAAGC
MAP1LC3A	AACATGAGCGAGTTGGTCAAG	GCTCGTAGATGTCCGCGAT
MAP1LC3B	GATGTCCGACTTATTCGAGAGC	TTGAGCTGTAAGCGCCTTCTA
MAPK8	TCTGGTATGATCCTTCTGAAGCA	TCCTCCAAGTCCATAACTTCCTT
MTOR	ATGCTTGGAACCGGACCTG	TCTTGACTCATCTCTCGGAGTT
NFKB1	GAAGCACGAATGACAGAGGC	GCTTGGCGGATTAGCTCTTTT
NPC1	ATCAGTGCCTAATTCAACACGC	GGAACTAACATCAGTTGCCAGAA
P53	CAGCACATGACGGAGGTTGT	TCATCCAAATACTCCACACGC
P62	AAGCCGGGTGGGAATGTTG	CCTGAACAGTTATCCGACTCCAT
PARK	AAGAGGGTGTTCTCTATGTAGGC	GCTCCTCCAACATTTGTCACTT
PI3K	GGCGAAACGCCCATCAAAAA	GACTCCCGTGCAGTCATCC
PIK3C3	CCTGGAAGACCCAATGTTGAAG	CGGGACCATACACATCCCAT
PTEN	TGGATTCGACTTAGACTTGACCT	GGTGGGTTATGGTCTTCAAAAGG
RAB24	TACGTGGGCAAGACTAGCCT	GCCCCGATGGTGTTCTGATAAG

RAB5A	CTGGTCAAGAACGATACCATAGC	GTCGGCCTTGTTTCCCGAT
RAB7A	GTGTTGCTGAAGGTTATCATCCT	GCTCCTATTGTGGCTTTGTACTG
Rgs9	GGCGCAGTCTTTTGACAAGC	GCCTTCTCGTCTACCACATGC
Rt-actin	TTCCAGCCTTCCTTCCTGGG	TTGCGCTCAGGAGGAGCAAT
Rt-AtgL	GGCTTCCTCGGCGTCTACTA	TTTACCAGGTTGAAGGAGGGG
SNCA	AAGAGGGTGTTCTCTATGTAGGC	GCTCCTCCAACATTTGTCACTT
TGFB1	CTAATGGTGGAAACCCACAACG	TATCGCCAGGAATTGTTGCTG
TGM2	GAGGAGCTGGTCTTAGAGAGG	CGGTCACGACACTGAAGGTG
TNF	CCTCTCTCTAATCAGCCCTCTG	GAGGACCTGGGAGTAGATGAG
TRAF6	ATGCGGCCATAGGTTCTGC	TCCTCAAGATGTCTCAGTTCCAT
ULK1	GGCAAGTTCGAGTTCTCCCG	CGACCTCCAAATCGTGCTTCT
ULK2	TGGAGACCTCGCAGATTATTTGC	CTGTGCAGGATTCGCATGG
UVRAG	GGCGTCTTCGACATCTTCGG	GACGGTCTGGCATAATTCCAAA
VPS16	TACACGGCGAACTGGAACC	GCCTCACACTAGCAGCTTTCT
WIPI1	ACTAAAGCCGGGTATAAGCTGT	CGGGATTTCATTGCTTCCGTG
WIPI2	CCATCGTCAGCCTTAAAGCAC	TCCAGGCATACTATCAGCCTC

Table S3. Clinical characteristics of NPC patients

**Characteristics**	**Number of patients (%)**
Gender	
Male	16 (59.3%)
Female	11 (40.7%)
Age	
≥45	12 (44.4%)
< 45	15 (55.6%)
Clinical Stage	
I	1 (3.7%)
II~III	8 (29.6%)
IV	18 (66.7%)
Therapeutic modality	
Radiation therapy	21 (77.8%)
Concomitant chemoradiaotherapy	11 (40.7%)
None treatment	1 (3.7%)
Histological classification	
NKUC	25 (92.6%)
KSCC	2 (7.7%)
EBER (In situ hybridization, ISH)	
positive	24(88.9%)
negative	3(11.1%)

NPC, nasopharyngeal carcinoma; NKCC, non-keratinizing carcinoma; KSCC, keratinizing squamous cell carcinoma. EBER, EBV-encoded RNA
